# The protective effects of lipoxin A4 on type 2 diabetes mellitus: A Chinese prospective cohort study

**DOI:** 10.3389/fendo.2023.1109747

**Published:** 2023-01-19

**Authors:** Sudan Wang, Xiaoyan Qian, Chao Shen, Qian Sun, Yang Jing, Bingyue Liu, Kexin Zhang, Mengyuan Li, Junrong Wang, Hui Zhou, Chen Dong

**Affiliations:** ^1^ Department of Epidemiology and Statistics, School of Public Health, Medical College of Soochow University, Soochow, Jiangsu, China; ^2^ Division of non-communicable diseases, Suzhou Industrial Park Centers for Disease Control and Prevention, Soochow, China

**Keywords:** type 2 diabetes mellitus, lipoxin A4, mediation analysis, cohort study, protective effects

## Abstract

**Background:**

Several cellular and animal studies have suggested that lipoxin A4 (LXA4) has a protective effect on type 2 diabetes mellitus (T2DM) development. However, little is known about whether LXA4 influences T2DM development at the population level.

**Methods:**

We included 2755 non-diabetic participants from a cohort study in China who were followed for about seven years. Cox proportional hazards model was used to estimate hazard ratios (HRs) and 95% confidence intervals (CI) for the association between LXA4 and incident T2DM. Mediation models were used to examine how serum lipids as mediators impact the association between LXA4 and T2DM.

**Results:**

In total, 172 newly diagnosed T2DM cases were identified. Multivariate-adjusted HR for T2DM in the fourth compared with the first quartile of LXA4 was 0.62 (95% CI: 0.40-0.96). When used the optimal cutoff value determined by the receiver operating characteristic curve, the results showed participants with LXA4 > 2.84 ng/mL had a decreased T2DM risk compared to those with LXA4 ≤ 2.84 ng/mL (HR: 0.63, 95% CI: 0.45-0.89). The effect of LXA4 on incident T2DM was significantly modified by gender (*P*_-interaction_ = 0.024) and family history of diabetes (*P*_-interaction_ = 0.025). Additionally, the association between LXA4 and incident T2DM was partially suppressed by the TyG and TG/HDL-c ratio, with a suppression proportion of 22.2% and 16.0%, respectively.

**Conclusions:**

Higher LXA4 levels are significantly associated with a lower risk of T2DM development. The present findings would be helpful in understanding the effect of LXA4 on T2DM development at the population level.

## 1 Introduction

Lipoxin A4 (LXA4), a member of the lipoxin family, is generated in human leukocytes *via* 15-lipoxigenase from arachidonic acid (1). As one of important specialized pro-resolving lipid mediators (SPMs), LXA4 acts as a critical brake after injury or infection *in vitro* and *in vivo* by inhibiting immune cell recruitment, chemotaxis, adhesion and transmigration (2). Based on recent studies, LXA4 can reduce nuclear factor-κB activation and the release of inflammatory cytokines such as tumor necrosis factor alpha (TNF-α) and high sensitivity C-reactive protein (hs-CRP) (3, 4). In addition, LXA4 and related SPMs may contribute to the biology of chronic diseases by acting as a balancing fulcrum between adipose inflammation and inflammatory resolution (5–7). In animal models, LXA4 exhibited the ability to reverse the adipose autophagy induced by a high-fat diet (8), suggesting that LXA4 represents a potentially novel therapeutic strategy for obesity-induced disease.

Over the last decade, several cellular and animal studies have suggested that LXA4 may have a direct effect on the adipocyte insulin signaling pathway, thereby preventing the development of type 2 diabetes mellitus (T2DM). For example, Barhina et al. reported that a significant reduction in plasma LXA4 levels in Wistar rats induced to develop T2DM by streptozotocin (STZ) (9). Moreover, LXA4 prevents the cytotoxic action of alloxan, doxorubicin, and benzo(a)pyrene and STZ against rat pancreatic β cells and possibly other normal cells (10). In addition, Gundala et al. reported that LXA4 production in RIN5 F cells and plasma LXA4 levels in STZ-induced T2DM animals were reduced, which returned to normal after arachidonic acid treatment (11). In a pilot clinical study involving 39 T2DM patients, Gutierrez et al. found that pioglitazone (15 or 30 mg/day) could increase 15-epi-LXA4 and adiponectin levels in the absence of significant changes in body weight (12).

In a cohort study involving 624 subjects aged 40-65 years, Yu et al. reported that LXA4 could be a protective factor and its decrease in serum might be a predictive biomarker of obesity-associated inflammation and early metabolic syndrome (13). However, little is known about whether, or to what extent, LXA4 influences T2DM development at the population level. Thus, in this study, we tested whether plasma LXA4 level is associated with the future T2DM risk in a well-established Chinese cohort study. The potentially effects of circulating lipid factors on the association between LXA4 and T2DM were also analyzed.

## 2 Methods

### 2.1 Study population

“The prevention of MS and multi-metabolic disorders in Jiangsu province of China II (PMMJS-II)” is an ongoing longitudinal study to explore the epidemiology of common metabolic disorders (i.e. T2DM, hypertension, obesity, metabolic syndrome, and cardiovascular diseases) and causes of death in community residents aged 35-60 years in Soochow, China. Details of the design and objectives of PMMJS-II have been described elsewhere (14). In Brief, a total of 3700 participants (1480 men and 2220 women) were recruited from four communities in Soochow between 06/2014 and 05/2015. Followed-up surveys were conducted every two years from baseline. Exclusion criteria for the PMMJS-II were (i) < 35 years or > 60 years; (ii) did not provide written informed consent. After further excluding individuals (i) with cancer, T2DM, chronic kidney disease, and chronic enteritis; (ii) with chronic viral hepatitis and chronic diarrhea; and (iii) had taken antibiotic, probiotic, or prebiotic products two months before the sample collection. 2755 participants were eventually included in the present analysis (1059 men and 1696 women) (**Figure S1**). The study protocol was approved by the Ethics Committee of Suzhou Industrial Park Centers for Disease Control and Prevention (Soochow, China), and conducted in accordance with the Declaration of Helsinki. Informed consent was obtained from all participants.

### 2.2 Assessment of plasma LXA4 concentration

Peripheral blood sample was taken from each participant after overnight fasting at baseline and at each follow-up. Thereafter, blood was centrifuged at 3000×g for 10 min and the separated plasma was frozen at -80°C immediately. Plasma LXA4 concentration was assessed using a human LXA4 enzyme linked immunosorbent assay (ELISA) kit (Elabscience Biotechnology Co., Ltd, Wuhan, China). The range of the LXA4 assay was 0.78-50 ng/mL based on manufacturing standards. Both intra and inter-assay coefficients were ≤ 10%.

Participants were divided by quartiles (Q1-4) of the LXA4 at baseline: ≤ 1.57 ng/mL, 1.57 to 2.24 ng/mL, 2.24 to 3.33 ng/mL, and > 3.33ng/mL. LXA4 level of 2.84 ng/mL was the optimal cutoff value for predicting T2DM, determined by the receiver operating characteristic (ROC) curve.

### 2.3 Assessment of T2DM diagnoses

All participants without a prior history of diabetes were followed until incident T2DM, death or the end of follow-up (31 December 2021), whichever was the earliest. Diagnosis of T2DM was made based on established criteria (ICD-10 code E11; http://apps.who.int/classifications/icd10/browse/2016/en) as following: (1) Random blood glucose levels ≥ 11.1mmol/L (200 mg/dL), (2) Fasting plasma glucose (FPG) levels ≥ 7.0 mmol/L (126 mg/dL), (3) 2-hour post-load glucose levels ≥ 11.1mmol/L, (4) Patients diagnosed with T2DM in secondary hospitals or above, (5) Gestational diabetes and other special types of diabetes were excluded. For those who did not provide the follow-up plasma samples, T2DM identification was based on the annual medical records and reviewed by a senior physician.

### 2.4 Assessment of covariates

Sociodemographic and lifestyle characteristics were collected at baseline by trained interviewers. These included information on gender, age, alcohol consumption, smoking, dietary habits, family history of diabetes, physical activity and medical history (e.g., dyslipidemia, or current medications). Men with alcohol intake > 40 g per day and women with alcohol intake > 30 g per day were classified as alcohol consumption. Physical activity was divided into weekly exercise time ≥ 4 hours and < 4 hours. The anthropometrics of each participant (e.g., blood pressure, height, weight) were measured by trained review staff. Hypertension was defined at recruitment as systolic blood pressure (SBP) ≥ 140 mmHg, diastolic blood pressure (DBP) ≥ 90 mmHg, antihypertensive medication use or prior diagnosis of hypertension. The concentrations of plasma total cholesterol (TC), triglycerides (TG), high-density lipoprotein cholesterol (HDL-c), FPG, and high-sensitivity C-reactive protein (hs-CRP) were determined at the central laboratory of the Suzhou Industrial Park Centers for Disease Control and Prevention. The low-density lipoprotein cholesterol (LDL-c) level was calculated using the Friedewald equation. TyG index was retrospectively calculated using the following formulas: TyG = Ln [fasting TG (mg/dL) × FPG (mg/dL)/2] (15). Dyslipidemia was defined as TG ≥ 1.7 mmol/L and/or TC ≥ 5.18 mmol/L and/or LDL-c ≥ 3.37 mmol/L and/or HDL-c ≤ 1.04 mmol/L, or use of lipid-lowering drugs.

### 2.5 Statistical analysis

Participants were divided into quartiles according to LXA4 concentrations. Baseline characteristics in each quartile were presented as means ± SD or medians (interquartile range [IQR]) for continuous variables and percentages for categorical variables, respectively. Differences between LXA4 quartiles were determined with χ^2^ test for categorical variables, whereas the variance analysis or Kruskal-Wallis test for continuous variables, as appropriate. The person-time of each participant was calculated using the time from the baseline visit to the end of follow-up on December 31, 2021, death, or time of diagnosis of T2DM, whichever came the first. We used Pearson correlation or Spearman’s test to examine the correlation between LXA4 and T2DM-related risk factors (e.g. age, BMI, TC, TG, HDL-c and LDL-c). In addition, the Kaplan-Meier curve was used to plot the incidence of T2DM across the LXA4 the optimal cutoff points determined by ROC analysis.

Cox proportional hazards models were used to calculate the hazard ratio (HR) and 95% confidence interval (CI) to estimate association between quartiles of LXA4 concentration and T2DM risk, after adjusting for potential confounders, including age, gender, BMI (kg/m^2^), smoking (yes/no), alcohol consumption (yes/no), hypertension (yes/no), dyslipidemia (yes/no), hs-CRP (mg/L), family history of diabetes (yes/no) and physical activity (yes/no). We used the lowest LXA4 quartile (< 1.57 ng/mL) as the reference group, and comparing LXA4 > 2.84 ng/mL to ≤ 2.84 ng/mL. To explore the possible dose-response relationships between LXA4 and incident T2DM, restricted cubic splines (RCS) was performed by trimming the highest and lowest 0.5% of LXA4 with knots placed at the 5th, 35th, 65th, and 95th percentile. In sensitivity analysis, we excluded the participants who used lipid-lowering drugs, with hs-CRP more than 5 mg/L and T2DM was developed in 2016. In addition, the stability of the association was verified by changing the BMI to WHR as the adjusted variables. In this study, we further evaluated the association between LXA4 and T2DM probability stratifying by age, gender, BMI, smoking, alcohol consumption, hypertension, dyslipidemia and family history of diabetes. Interactions between plasma LXA4 concentration and subgroup variables on T2DM probability were assessed by ANOVA using models with interaction terms, adjusted for the aforementioned covariates, unless the variable was used as a subgroup variable.

Given the significant impact of circulating lipids on the risk of T2DM, mediation models were used to examine whether the association of the LXA4 with T2DM risk was mediated by plasma lipid (TC, TG, HDL-c, LDL-c) or its related index (TyG and TG/HDL-c ratio). The LXA4 was exposure variable (X); risk factors above mentioned were mediators (M); incident T2DM was outcome variable (Y), following a binomial distribution. To enable comparison of the effect sizes of the different mediating factors, all mediating variable were transformed into Z-score before mediation analysis. The mediation effect strength was estimated by calculating the absolute value of the ratio of indirect to direct effects (16). Mediation analysis was performed using R package lavaan.

All the analyses were performed using the statistical package R (http://www.R-project.org, The R Foundation), and a two-sided *P* < 0.05 was considered to be statistically significant.

## 3 Results

The baseline characteristics of participants stratified by quartiles of LXA4 concentrations are presented in **Table 1**. The participants with higher levels of LXA4 appeared to be younger, nonsmokers and less likely to be with hypertension. The proportion of males in the quartile 1 and 4 groups was 42.8% and 33.3% (*P* = 0.004), respectively. In addition, participants with the highest quartile of LXA4 were more likely to have higher levels of TG, hs-CRP, TyG, and TG/HDL-c ratio (**Table 1**). As shown in **Table S1**, the plasma LXA4 concentration was negatively correlated with age, and positively correlated with TG, hs-CRP, TyG index and TG/HDL-c ratio (all *P* < 0.05).

**Table 1:** Baseline characteristics of the study participant according to the quartiles of LXA4.

Variables	LXA4 (ng/mL)				*P*
	Q1 (≤ 1.57)	Q2 (1.57-2.24)	Q3 (2.24-3.33)	Q4 (> 3.33)	
Age (years)	50.46 ± 5.98	49.96 ± 5.90	49.56 ± 6.05	49.69 ± 6.04	**0.026**
Male, n (%)	295 (42.8)	264 (39.1)	265 (38.7)	235 (33.3)	**0.004**
BMI (kg/m^2^)	23.97 ± 3.11	23.63 ± 2.78	23.81 ± 3.00	23.94 ± 2.99	0.124
WHR	0.88 ± 0.07	0.88 ± 0.08	0.88 ± 0.27	0.87 ± 0.07	0.717
SBP (mmHg)	124.72 ± 15.29	123.52 ± 15.56	123.51 ± 15.47	123.80 ± 14.67	0.420
DBP (mmHg)	78.01 ± 11.58	77.14 ± 11.22	77.31 ± 11.46	77.40 ± 11.06	0.527
Smoking, n (%)	194 (28.2)	180 (26.6)	174 (25.4)	150 (21.3)	**0.022**
Alcohol consumption, n (%)	138 (20.0)	126 (18.6)	134 (19.6)	106 (15.0)	0.066
Physical activity ≥ 4h/week	220 (31.9)	206 (30.5)	199 (29.1)	242 (34.3)	0.179
Family history of DM, n (%)	85 (12.3)	72 (10.7)	96 (14.0)	88 (12.5)	0.313
Hypertension, n (%)	283 (41.1)	230 (34.0)	266 (38.8)	283 (40.1)	**0.037**
Dyslipidemia, n (%)	226 (32.8)	213 (31.5)	225 (32.8)	250 (35.5)	0.460
Lipid-lowering drugs, n (%)	7 (1.0)	2 (0.3)	4 (0.6)	6 (0.9)	0.394
FPG (mmol/L)	5.49 ± 0.42	5.47 ± 0.39	5.46 ± 0.40	5.46 ± 0.39	0.478
TC (mmol/L)	4.90 ± 0.93	4.80 ± 0.87	4.84 ± 0.90	4.87 ± 0.90	0.213
TG (mmol/L)	1.40 ± 0.96	1.37 ± 0.82	1.49 ± 1.06	1.57 ± 1.17	**0.001**
HDL-c (mmol/L)	1.24 ± 0.31	1.23 ± 0.27	1.22 ± 0.28	1.23 ± 0.31	0.502
LDL-c (mmol/L)	3.07 ± 0.75	2.98 ± 0.71	3.01 ± 0.72	3.03 ± 0.78	0.153
hs-CRP (mg/L)	1.23 (0.94-1.84)	1.21 (0.93-1.87)	1.24 (0.94-1.84)	1.32 (0.99-2.02)	**0.022**
LXA4 (ng/mL)	1.22 (0.97-1.41)	1.90 (1.73-2.06)	2.69 (2.45-2.94)	4.78 (3.91-7.43)	**< 0.001**
TyG	8.56 ± 0.54	8.55 ± 0.52	8.61 ± 0.56	8.64 ± 0.59	**0.010**
TG/HDL-c	2.20 (1.40-3.53)	2.20 (1.44-3.45)	2.32 (1.51-3.80)	2.32 (1.51-3.80)	**0.033**

Data are presented as means ± SD, medians (IQR), or n(%).

Significant data in the table are in bold.

During a follow-up period of seven years, 172 new T2DM cases were documented. The incidence density was 10.09 per 1000 person-years. When comparing with the participants in the first quartile of the LXA4, the risk of T2DM was significantly decreased in those in the fourth quartile (HR = 0.61, 95% CI: 0.39-0.94) (model 1). After adjusting for age, gender, and other potential confounders (model 3), the association between LXA4 and T2DM probability remained significant for the highest *vs* the first quartile of the plasma LXA4 level (HR = 0.62, 95% CI: 0.40-0.96) (**Table 2**). In Models 1-3, participants with LXA4 > 2.84 ng/mL exhibited a reduced risk of T2DM when compared to those with LXA4 ≤ 2.84 ng/mL (HR = 0.63, 95% CI: 0.45-0.89) (**Table 2**). The Kaplan-Meier survival plot stratifying LXA4 into low (≤ 2.84 ng/mL) versus high (> 2.84 ng/mL) levels showed similar results (**Figure S2**, *P* = 0.003). As the results shown in **Table 3**, the association between LXA4 and T2DM probability remained stable even after excluding participants who used lipid-lowering drugs, with elevated hs-CRP level (> 5 mg/L), new-onset T2DM in 2016, and changing adjustment variables BMI to WHR. In addition, the results of RCS showed a nonlinear dose-response association between LXA4 and T2DM probability (**Figure S3**, *P*_linearity_ = 0.085).

**Table 2:** Association between LXA4 and the incidence of T2DM.

	N (case)	Model 1	*P* value	Model 2	*P* value	Model 3	*P* value
		HR (95% CI)		HR (95% CI)		HR (95% CI)	
LXA4 quartiles							
Q 1	689 (52)	Ref	1.0	Ref	1.0	Ref	1.0
Q 2	676 (41)	0.79 (0.52-1.19)	0.256	0.84 (0.55-1.26)	0.389	0.90 (0.60-1.36)	0.620
Q 3	685 (46)	0.88 (0.59-1.31)	0.529	0.92 (0.62-1.37)	0.671	0.90 (0.60-1.35)	0.615
Q 4	705 (33)	0.61 (0.39-0.94)	**0.026**	0.64 (0.41-0.99)	**0.046**	0.62 (0.40-0.96)	**0.031**
*P _trend_*	0.052		0.051		0.081		0.045
LXA4 > 2.84 ng/mL	942 (44)	0.66 (0.47-0.92)	**0.016**	0.68 (0.48-0.95)	**0.025**	0.63 (0.45-0.89)	**0.009**
Per 1-SD increase		0.77 (0.51-1.16)	0.215	0.79 (0.53-1.17)	0.240	0.78 (0.51-1.18)	0.239

Model 1: unadjusted.

Model 2: adjusted for baseline age, gender, BMI, smoking, alcohol consumption.

Model 3: adjusted for baseline age, gender, BMI, smoking, alcohol consumption, hypertension, dyslipidemia, hs-CRP, family history of diabetes and physical activity.

Significant data in the table are in bold.

**Table 3:** Sensitivity analysis of the association between LXA4 and the incidence of T2DM.

	N (case)	Model 1	*P* value	Model 2	*P* value	Model 3	*P* value
		HR (95% CI)		HR (95% CI)		HR (95% CI)	
Excluding participants with hs-CRP > 5 mg/L							
Q 1	671 (52)	Ref	1.0	Ref	1.0	Ref	1.0
Q 2	655 (39)	0.75 (0.50-1.14)	0.181	0.80 (0.52-1.21)	0.279	0.85 (0.56-1.29)	0.447
Q 3	666 (43)	0.82 (0.55-1.23)	0.346	0.86 (0.57-1.29)	0.453	0.82 (0.55-1.24)	0.346
Q 4	678 (31)	0.58 (0.37-0.90)	0.016	0.61 (0.39-0.95)	0.030	0.58 (0.37-0.91)	0.018
*P _trend_*	0.029		0.029		0.049		0.022
LXA4 > 2.84 ng/mL	909 (41)	0.63 (0.45-0.90)	0.011	0.65 (0.46-0.93)	0.018	0.60 (0.42-0.85)	0.005
Per 1-SD increase		0.77 (0.51-1.18)	0.229	0.79 (0.53-1.18)	0.252	0.77 (0.50-1.18)	0.230
Excluding participants using lipid-lowering drugs							
Q 1	689 (51)	Ref	1.0	Ref	1.0	Ref	1.0
Q 2	679 (40)	0.78 (0.52-1.18)	0.243	0.82 (0.54-1.25)	0.356	0.88 (0.58-1.34)	0.550
Q 3	678 (47)	0.93 (0.62-1.38)	0.711	0.96 (0.65-1.44)	0.858	0.95 (0.63-1.41)	0.785
Q 4	690 (29)	0.56 (0.35-0.88)	0.012	0.58 (0.37-0.92)	0.020	0.56 (0.35-0.89)	0.013
*P _trend_*	0.035		0.035		0.053		0.029
LXA4 > 2.84 ng/mL	934 (41)	0.62 (0.44-0.88)	0.008	0.64 (0.45-0.91)	0.013	0.60 (0.42-0.85)	0.004
Per 1-SD increase		0.75 (0.48-1.16)	0.197	0.77 (0.51-1.17)	0.221	0.76 (0.49-1.18)	0.220
Excluding participants diagnosed with T2DM in 2016							
Q 1	684 (41)	Ref	1.0	Ref	1.0	Ref	1.0
Q 2	683 (33)	0.79 (0.50-1.25)	0.313	0.83 (0.53-1.32)	0.429	0.91 (0.57-1.44)	0.673
Q 3	671 (41)	1.01 (0.66-1.56)	0.958	1.04 (0.67-1.61)	0.859	1.04 (0.67-1.62)	0.850
Q 4	682 (22)	0.53 (0.31-0.88)	0.015	0.55 (0.33-0.92)	0.024	0.53 (0.31-0.89)	0.016
*P _trend_*	0.060		0.058		0.079		0.047
LXA4 > 2.84 ng/mL	929 (31)	0.56 (0.37-0.83)	0.004	0.57 (0.38-0.86)	0.007	0.53 (0.35-0.79)	0.002
Per 1-SD increase		0.37 (0.16-0.86)	0.020	0.40 (0.18-0.91)	0.028	0.37 (0.16-0.86)	0.021
Changing the adjustment variable BMI to WHR							
Q 1	689 (52)	Ref	1.0	Ref	1.0	Ref	1.0
Q 2	676 (41)	0.79 (0.52-1.19)	0.256	0.80 (0.53-1.22)	0.299	0.89 (0.58-1.35)	0.571
Q 3	685 (46)	0.88 (0.59-1.31)	0.529	0.90 (0.60-1.35)	0.607	0.91 (0.60-1.36)	0.631
Q 4	705 (33)	0.61 (0.39-0.94)	0.026	0.66 (0.43-1.02)	0.062	0.64 (0.41-0.99)	0.046
*P _trend_*	0.052		0.051		0.108		0.067
LXA4 > 2.84 ng/mL	942 (44)	0.66 (0.47-0.92)	0.016	0.68 (0.48-0.97)	0.031	0.64 (0.45-0.90)	0.011
Per 1-SD increase		0.77 (0.51-1.16)	0.215	0.80 (0.55-1.18)	0.268	0.79 (0.53-1.19)	0.265

Model 1: unadjusted.

Model 2: adjusted for baseline age, gender, BMI (WHR), smoking, alcohol consumption.

Model 3: adjusted for baseline age, gender, BMI (WHR), smoking, alcohol consumption, hypertension, dyslipidemia, hs-CRP, family history of diabetes and physical activity.

**Figure 1:**
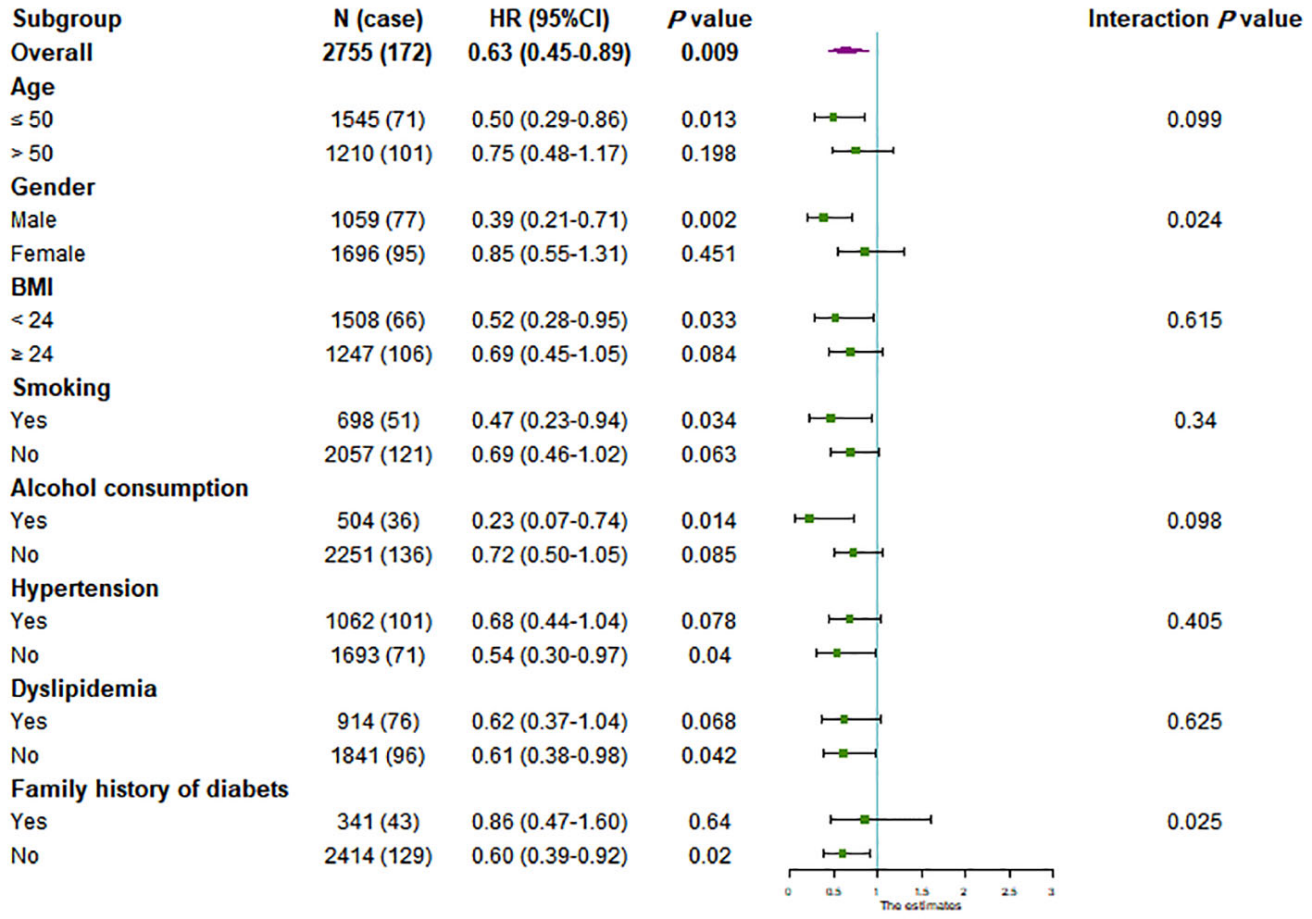
Subgroup analysis of the association between LXA4 and the incident of T2DM. Cox regression after adjustment for baseline age, gender, BMI, smoking, alcohol consumption, hypertension, dyslipidemia, hs-CRP, family history of diabetes and physical activity was performed in subgroups according to age (≤ 50 or > 50 years), gender (male or female), body mass index (BMI; < 24 or ≥ 24 kg/m2), smoking (yes or no), alcohol consumption (yes or no), hypertension (yes or no), dyslipidemia (yes or no), and family history of diabetes (yes or no).

We further conducted subgroup analyses stratified by gender, age, BMI, smoking, alcohol consumption, hypertension, dyslipidemia and family history of diabetes to estimate the association between LXA4 and future T2DM probability. Compared with the participants with LXA4 ≤ 2.84 ng/mL, the high-level group of LXA4 (> 2.84ng/mL) was significantly associated with a lower risk ofT2DM in the subgroup of age ≤ 50-year, male, BMI < 24 kg/m^2^, smoking, alcohol consumption, normotension, normolipidemia and the participants without family history of diabetes (**Figure 1**). Moreover, the effect of LXA4 on the outcome of T2DM was significantly modified by gender (*P*_-interaction_ = 0.024) and family history of diabetes (*P*_-interaction_ = 0.025). The association was stronger in males (HR: 0.39, 95% CI: 0.21-0.71) and individuals without family history of diabetes (HR: 0.60, 95% CI: 0.39-0.92).

**Figure 2:**
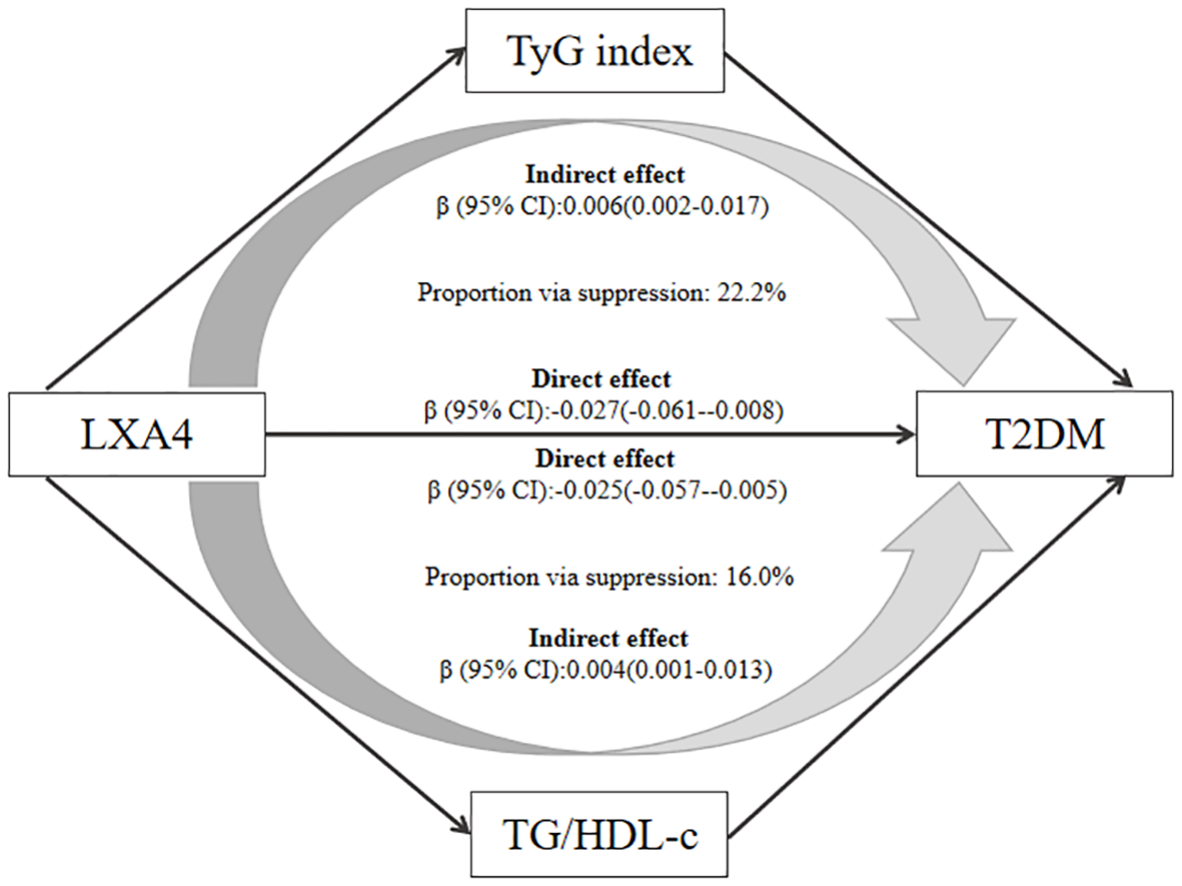
The mediating role of TyG and TG/HDL-c in the association between LXA4 and T2DM. All mediators were standardized using Z-scores to facilitate comparison. Adjusted for baseline age and gender.

Furthermore, we tested which lipid factors (TC, TG, HDL-c, LDL-c, and lipid-associated index of TyG and TG/HDL-c) mediated the association between LXA4 and the outcome of T2DM. The results showed that the association of LXA4 with T2DM was partially suppressed by TyG and TG/HDL-c ratio after adjustment for age and gender, with a suppression proportion of 22.2% for TyG index and 16.0% for TG/HDL-c ratio, respectively (**Figure 2**). However, TC, TG, HDL-c and LDL-c did not significantly mediate the association between LXA4 and future T2DM probability (**Table S2**).

## 4 Discussion

In this study, we reported that there is a correlation between higher LXA4 level and lower risk of T2DM. In addition, the effect of LXA4 on T2DM incidence was modified by gender and family history of diabetes. Moreover, the mediation models suggested that TyG and TG/HDL-c ratio could partially suppress the prospective association between LXA4 and T2DM. The findings of our study are consistent with evidence from previous cellular studies and animal models. For example, Börgeson et al.reported that LXA4 could reduce inflammation by affecting both macrophages and adipocytes in high-fat diet-induced mice models. Moreover, LXA4 could shift the macrophage phenotype from M1 to M2 *in vitro* (17). In addition, LXA4 significantly reduced macrophages secretion of the proinflammatory cytokine TNF-ɑ, which likely presents the mechanism by which glucose uptake was rescued, since TNF-ɑ is one of the crucial cytokines in adipose insulin insistence and inhibits glucose uptake (18). Thus, our present results complement and extend those from prior studies that examined the protective effect of LXA4 on the subsequent risk of T2DM development.

In this community-based study, we observed an interaction between gender and LXA4 on the probability of future T2DM. It has been reported that there are gender differences in the expression and activity of synthetases (such as 5-lipoxygenase, 12-lipoxygenase and 15-lipoxygenase) for LXA4 production in animal models (19, 20). Compared to female mice, the male mice could produce more LXA4 from omega-6 fatty acids (21). On the other hand, increasing evidence has suggested that gender could affect the glucose homeostasis and the pathophysiology, incidence and prevalence of T2DM (22–24). In a large study population of 1,462 Danish adults, Faerch et al. reported that the increase in serum GLP-1 concentration following an OGTT was greater in normoglycemic females than in normoglycemic males (25). In addition, Horst et al. reported that adipose tissue inflammation was positively correlated with circulating leptin and IL-6 only in males, and was negatively correlated with adipose tissue score in females (26). Taken together, the evidence from previous studies may partially explain our present findings.

Furthermore, our present study suggested that the effect of LXA4 on T2DM incidence was modified by family history of diabetes. Compared with participants in the lower LXA4 concentration group, those in the higher LXA4 concentration group had a 40% lower risk of T2DM in the participants without family history of diabetes, but only a 14% reduction in the participants with family history of diabetes. Interaction between LXA4 and family history of diabetes might be hypothesized to be caused by genetic factors. Based on the clinical study LIMEX, Koc et al. proposed that the expression of genes involved in the control of inflammation and lipid metabolism in monocytes and lymphocytes was altered in response to glucose uptake in first-degree relatives of T2DM patients compared to controls (27). Given that LXA4 concentrations were significantly lower in participants with family history of diabetes than in those without family history of diabetes, the impact of genetic predisposition on the association between LXA4 and T2DM risk should be an interesting and meaningful question for future research.

TyG index, calculated from fasting glucose and triglycerides, has been proposed as a reliable marker of insulin resistance (28–30). In a Singapore cohort study involving 4109 participants, Low et al. reported that TyG was significantly associated with a higher risk of developing T2DM. Moreover, TyG mediates about 35% of the effect of BMI on the risk of T2DM (31). Similarly, the ratio of TG/HDL-c is a surrogate marker of insulin resistance and is more convenient, economical, and available than the gold standard for insulin resistance detection (32, 33). In a Japanese cohort study, Shimodaira et al. suggested that a 1-SD increase in LnTG/HDL-c ratio associated with > 12% increased risk of T2DM development (34). Therefore, it is not surprising to observe that both TyG index and TG/HDL-c ratio can suppress the impact of LXA4 on the future T2DM probability. However, given that TyG index and TG/HDL-c ratio are reliable measures of insulin resistance, the mediation effect of insulin on the association between LXA4 and the risk of T2DM development should be further elucidated.

The most important advantage of our study is that the association between LXA4 and T2DM has been explored for the first time in a prospective cohort study, providing new and strong evidence for the protective effect of LXA4 on the development of T2DM. In addition, a series of sensitivity analyses were carried out to improve the reliability of the results and to avoid chance in the data analysis. However, our study has several limitations. First, plasma LXA4 concentration may be influenced by the absolute amount of arachidonic acid in the diet. However, the detailed information on the relevant arachidonic acid consumption is not collected in this study. Considering that Han Chinese generally have similar dietary habits within the same region, the dietary effects may be very limited. Second, the plasma levels of LXA4 and other parameters were measured only at the baseline. The levels of these variables may have changed over time before the incidence of the disease. Therefore, the characteristic of LXA4 trajectory during the follow-up and its effect on the T2DM probability should be examined in the future. Third, our present results suggested that the protective effect of LXA4 on the risk of T2DM were strengthened in several subgroups, including the participants with age ≤ 50 years, BMI < 24 kg/m^2^, smoking, alcohol consumption, normotension, normolipidemia, suggesting that a complex effect of traditional T2DM risk factors on the association between LXA4 and T2DM risk. Finally, given that this prospective cohort study was conducted among Chinese adults and has a limited sample size, our present findings should be applied with caution to different population groups.

In summary, our findings provide novel evidence that LXA4 has a protective effect on the risk of T2DM development at the population level. However, the current findings need to be further supported by evidence at the level of gene or protein expression *in vivo* and *in vitro*. Moreover, given that the balance between the occurrence and resolution of inflammation is complicated, additional measurements of inflammatory-associated molecules and analyses of the effect of LXA4 interaction with these molecules on T2DM risk should be conducted in the future.

## Data availability statement

The raw data supporting the conclusions of this article will be made available by the authors, without undue reservation.

## Ethics statement

The studies involving human participants were reviewed and approved by Suzhou Industrial Park Centers for Disease Control and Prevention. The patients/participants provided their written informed consent to participate in this study.

## Author contributions

HZ and CD contributed to the conception and design of the study; SW, XQ, CS and CD contributed to manuscript drafting; SW, XQ, CS, QS, BL and ML contributed to the statistics analysis; YJ, HZ, KZ and JW contributed to the acquisition of data; SW, HZ and CD contributed to critical revisions of the manuscript. All authors contributed to the article and approved the submitted version.

## Supplementary material

Supplementary Figure 1 Flow chart of study population.

Supplementary Figure 2 Cumulative incidence of T2DM based on the optimal cutoff value of baseline LXA4.

Supplementary Figure 3 Association between baseline LXA4 and the risk of T2DM. Baseline LXA4 was assessed as a continuous variable using restricted cubic spline regression, adjusted for age, gender, BMI, smoking, alcohol consumption, hypertension, dyslipidemia, hs-CRP, family history of diabetes and physical activity. *P*-values for linearity: 0.085.
