# Supplementary figures and images for: The protective effects of lipoxin A4 on type 2 diabetes mellitus: A Chinese prospective cohort study
Source: Front Endocrinol (Lausanne). 2023 Jan 19;14:1109747. doi: 10.3389/fendo.2023.1109747 (PMC9892446; doi:10.3389/fendo.2023.1109747)

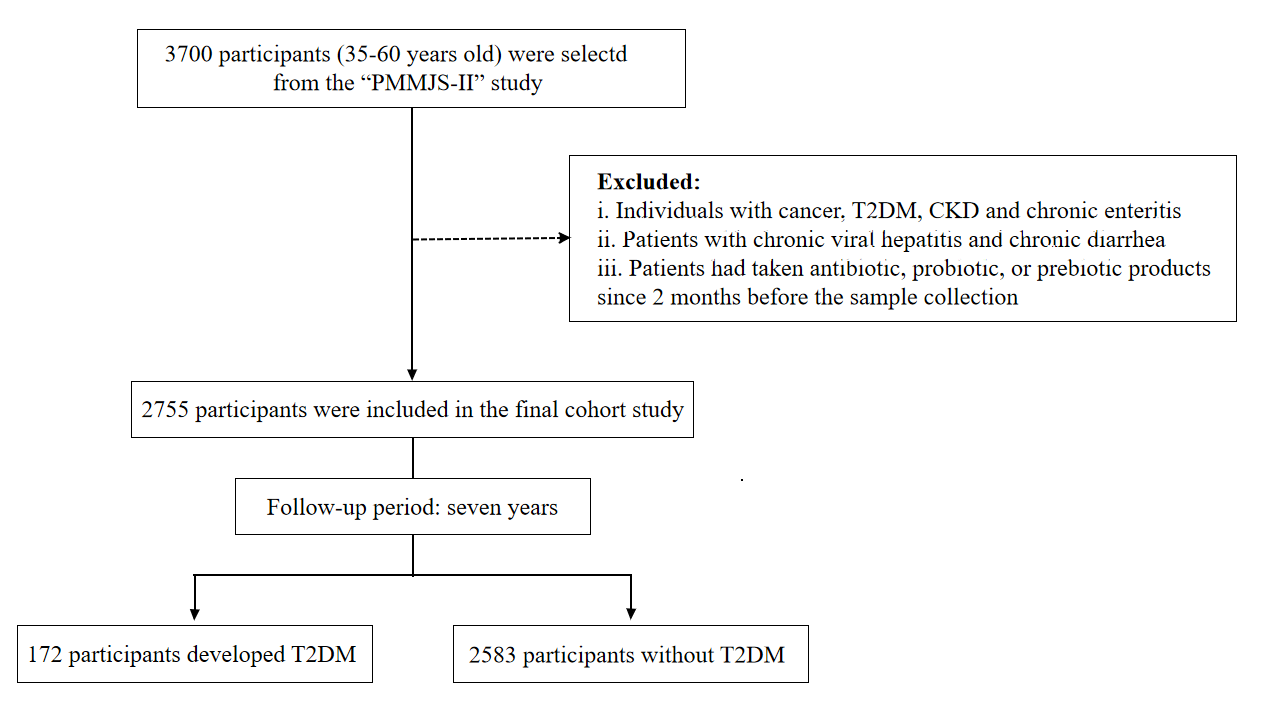

Supplement: Supplementary Figure 1 — Flow chart of study population. [file Image_1.tif]

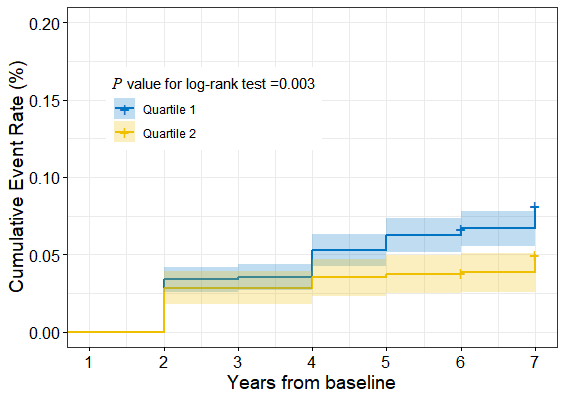

Supplement: Supplementary Figure 2 — Cumulative incidence of T2DM based on the optimal cutoff value of baseline LXA4. [file Image_2.tif]

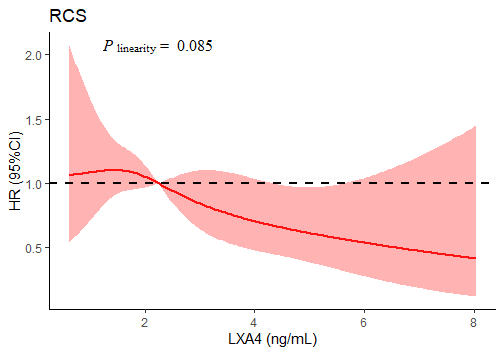

Supplement: Supplementary Figure 3 — Association between baseline LXA4 and the risk of T2DM. Baseline LXA4 was assessed as a continuous variable using restricted cubic spline regression, adjusted for age, gender, BMI, smoking, alcohol consumption, hypertension, dyslipidemia, hs-CRP, family history of diabetes and physical activity. P-values for linearity: 0.085. [file Image_3.tif]
