# Supplementary material for: The protective effects of lipoxin A4 on type 2 diabetes mellitus: A Chinese prospective cohort study
Source: Front Endocrinol (Lausanne). 2023 Jan 19;14:1109747. doi: 10.3389/fendo.2023.1109747 (PMC9892446; doi:10.3389/fendo.2023.1109747)
Supplement: Supplementary file 4 [file Table_1.docx]

Table S1 Linear association of LXA4 with traditional T2DM-related risk factors

| Variables | r | *P* |
| --- | --- | --- |
| Age | -0.054 | 0.005 |
| BMI | 0.004 | 0.835 |
| WHR | -0.022 | 0.258 |
| SBP | -0.014 | 0.476 |
| DBP | -0.016 | 0.408 |
| FPG | -0.029 | 0.126 |
| TC | 0.005 | 0.791 |
| TG | 0.057 | 0.003 |
| HDL | -0.012 | 0.536 |
| LDL | -0.009 | 0.652 |
| hs-CRP | 0.053 | 0.006 |
| TyG | 0.053 | 0.007 |
| TG/HDL | 0.055 | 0.005 |
