# Supplementary material for: The protective effects of lipoxin A4 on type 2 diabetes mellitus: A Chinese prospective cohort study
Source: Front Endocrinol (Lausanne). 2023 Jan 19;14:1109747. doi: 10.3389/fendo.2023.1109747 (PMC9892446; doi:10.3389/fendo.2023.1109747)
Supplement: Supplementary file 5 [file Table_2.docx]

Table S2 Mediation effects of TC, TG, HDL-c, and LDL-c on the association between LXA4 and T2DM

| Mediators^†^ | Indirect effect Estimate  (95% CI) | Direct effect Estimate  (95% CI) | Total effect Estimate  (95% CI) | Proportion via suppression % |
| --- | --- | --- | --- | --- |
| TC | 0.000 (-0.000-0.004) | -0.021 (-0.050--0.002)^*^ | -0.023 (-0.050--0.001)^*^ | -1.0 |
| TG | 0.003 (-0.000-0.009) | -0.024 (-0.056--0.004)^*^ | -0.023 (-0.050--0.001)^*^ | -12.5 |
| HDL-c | 0.001 (-0.003-0.003) | -0.022 (-0.049--0.001)^*^ | -0.023 (-0.050--0.001)^*^ | -4.6 |
| LDL-c | 0.000 (-0.001-0.004) | -0.021 (-0.050--0.001)^*^ | -0.023 (-0.050--0.001)^*^ | -3.3 |

^†^All mediators were standardized using Z-scores to facilitate comparison

^*^ *P* < 0.05

Adjusted for baseline age and gender.
